# *Bletilla striata* Micron Particles Function as a Hemostatic Agent by Promoting Rapid Blood Aggregation

**DOI:** 10.1155/2017/5820405

**Published:** 2017-03-12

**Authors:** Chen Zhang, Rui Zeng, Zhencheng Liao, Chaomei Fu, Hui Luo, Hanshuo Yang, Yan Qu

**Affiliations:** ^1^The Ministry of Education Key Laboratory of Standardization of Chinese Herbal Medicine, Key Laboratory of Systematic Research, Development and Utilization of Chinese Medicine Resources in Sichuan Province-Key Laboratory Breeding Base Co-Founded by Sichuan Province and MOST, Pharmacy College, Chengdu University of Traditional Chinese Medicine, Chengdu 611137, China; ^2^College of Pharmacy, Southwest University for Nationalities, Chengdu 610041, China; ^3^State Key Laboratory of Biotherapy and Cancer Center, West China Hospital, Sichuan University and Collaborative Innovation Center for Biotherapy, Chengdu, 610041, China

## Abstract

The human body cannot control blood loss without treatment. Available hemostatic agents are ineffective at treating cases of severe bleeding and are expensive or raise safety concerns.* Bletilla striata *serve as an inexpensive, natural, and promising alternative. However, no detailed studies on its hemostatic approach have been performed. The aim of this study was to examine the hemostatic effects of* B. striata* Micron Particles (BSMPs) and their hemostatic mechanisms. We prepared and characterized BSMPs of different size ranges and investigated their use as hemostatic agent. BSMPs of different size ranges were characterized by scanning electron microscope. In vitro coagulation studies revealed BSMP-blood aggregate formation via stereoscope and texture analyzers. In vivo studies based on rat injury model illustrated the BSMP capabilities under conditions of hemostasis. Compared to other BSMPs of different size ranges, BSMPs of 350–250 *μ*m are most efficient in hemostasis. As powder sizes decrease, the degree of aggregation between particles and hemostatic BSMP effects declines. The BSMP in contact with a bleeding surface locally forms a visible particle/blood aggregate as a physical barrier that facilitates hemostasis. Considering the facile preparation, low cost, and long shelf life of* B. striata*, BSMPs offer great potential as mechanisms of trauma treatment.

## 1. Introduction

Uncontrolled bleeding is a major cause of trauma-related death [1]. The human body's physiological response to injury involves three stages: plate plug formation and enzymatic cascade formation resulting in fibrin generation and clot dissolution and wound site healing [2, 3]. The human body's natural mechanisms cannot control massive amounts of blood loss resulting from major traumas [4]. Sustaining hemostasis in cases of clinical hemorrhaging is a challenging task that involves applying extensive efforts to stabilize medically difficult-to-treat traumatic injuries [5].

In general, an ideal hemostatic agent should be highly efficacious, easy to use and sterilize, nonantigenic, stable, and inexpensive [6].* B. striata *(Thunb.) Reichb. f. (Orchidaceae) known as Hyacinth Orchid, Common Bletilla Tuber, Japanorchidee (German), Shiran (Japanese), Jaran (Korean), and Baiji (baiji) is not only an ornamental garden in Europe and United States but an important astringent hemostatic medicinal plant native to East Asia [7, 8]. Traditional Chinese Medicine (TCM) holds that it is capable of restraining leakage of blood and stopping bleeding, dispersing swelling, and promoting tissue regeneration [9]. Thus, it could be effectively applied in the treatment of hematemesis, traumatic bleeding, and ulcerative carbuncle [10, 11].

These findings from traditional applications suggest that* Bletilla striata *particles can be used as hemostatic agents to treat traumatic bleeding. However, no detailed studies on their hemostatic modes of action have been performed. In a previous study, we found that particle size ranges are the main factor affecting hemostatic outcomes. To improve the hemostatic efficiency of BSMPs, explore its hemostatic mechanism, and determine the best size range for hemostasis, we characterized BSMPs into different size ranges (350–250, 250–180, 180–125, 125–75, and <75 *μ*m), by means of scanning electron microscope and Fourier transform infrared (FTIR) spectroscopy in conjunction with physical characterization measurements. We conducted in vivo efficacy studies on rats. Through in vitro blood/BSMP coagulation studies, internal characterizations of blood/BSMP aggregation based on texture analyzer stereoscope and physical property analyses were then used to assess the bioactivity and efficacy of BSMPs of different size ranges.

## 2. Materials and Methods

### 2.1. Materials

Tubers of* B. striata *were purchased from Sichuan Chinese Medicine Yinpian Co. Ltd. Sprague-Dawley (SD) rats were obtained from the Chengdu Dossy Experimental Animal Co. Ltd., China. All other chemicals were of analytical grade.

### 2.2. BSMP Preparation and Characterization

The plant material was oven-dried at 60°C for 24 h and finely pulverized using QE-300 g Omnipotent Disintegrator (Zhejiang Yili Garment Co., Ltd.) and Micronizing Pharmaceutical Vibrating Mill (Jinan Beili Co., Ltd.). Particles of different particle size ranges (350–250, 250–180, 180–125, 125–75, and <75 *μ*m) were strained through matched sieves. Morphological characterizations of BSMPs were performed on ZEISS SUPRA 40 (Germany) SEM at an accelerated voltage of 150 KV and at a working distance of 10–15 mm. The samples were coated with 10 nm thick platinum pieces to make the samples conductive. The specific surface area (m^2^/g) of BSMP was determined by measuring the adsorption of nitrogen according to the Brunauer-Emmett-Teller (BET) principle and using the ASAP 2010 instrument (Micromeritics instrument Co., USA). Measurements were repeated three times after degassing each sample for 24 h at 40°C. FTIR spectrum was obtained using a Spectrum One FTIR (PerkinElmer Co., USA). In brief, the BSMP samples were formed into pellets with KBr and then scanned under 4,000 to 400 cm^−1^ wavelengths. Five replicated spectra were collected for every sample pressed on the ATR crystal. The background spectrum was obtained against the air.

### 2.3. Blood/BSMP Aggregation

The dry BSMP was added in 1.5 ml EP tubes. Blood was collected via the abdominal aortic method from SD rats through vacuum pick blood vessels containing 10% (w/v) sodium citrate anticoagulant to prevent blood clotting. We then added 0.5 ml of anticoagulant blood to EP tube vials containing BSMPs. Vials were then rotated for 30 s and set vertically on the lab bench. The vials were inverted every 30 s until the blood/BSMPs completely ceased to flow, and the time period of this stage was recorded. All experimental groups were run in triplicate (*n* = 3).

### 2.4. Internal Structure of Blood/BSMP Aggregation

We covered 5 g of BSMP in glass garden (5 × 5 cm). Anticoagulant blood was added through a pipetting gun to the surfaces of the BSMPs to ensure blood scattering. After 5 minutes, surface characterizations of BSMP anticoagulant blood absorption were performed using images acquired from a Discovery.V20 from Zeiss stereoscope (Germany). BSMPs that absorbed the anticoagulant blood formed an aggregation. The blood/BSMP aggregation was embedded into Tissue Freezing Medium. A 10 *μ*m thick frozen section was then cut using a CM1520 from Leica Freezing Microtome (USA). The internal characterization of the frozen section was imaged using a Discovery.V20 from Zeiss stereoscope (Germany).

### 2.5. Texture Analysis of Blood/BSMP Aggregation

The blood/BSMP aggregations were then collected. We then conducted a texture analysis of the blood/BSMP aggregations using a Food Technology Corporation TMS-Pro Texture Analyzer (USA) fitted with a 250 N Intelligent Loadcell and a 6 mm diameter cylinder probe and programed to test a series of blood/BSMP aggregations. The test program moved the probe at 50 mm/min to meet the aggregation and then moved it an additional 2 mm to break it before returning to the starting position. The TMS-Pro software program was then used to analyze the data and to calculate the peak force achieved upon breaking each sample. The patterns of each breakage event were also assessed visually. A TMS-Pro graphical representation of the sample test results is shown here (force applied against cumulative displacement). All experimental groups were run in triplicate (*n* = 3).

### 2.6. Rat Tail Amputation

Hemostatic effects of BSMP in terms of stopping bleeding were evaluated using a tail amputation model and healthy male Sprague-Dawley (SD) rats (250 ± 20 g, 7 weeks of age). Rats were divided into six groups of five treated with cotton gauze and BSMPs (350–250, 250–180, 180–125, 125–75, and <75 *μ*m), respectively. Animal procedures were carried out under an institutionally approved protocol in accordance with ethical principles and standards of the Federation of European Animal Science Associations and were approved by the Ethical Committee at the Chengdu University of Traditional Chinese Medicine. All rats were anesthetized with 1.25 ml 10% chloral hydrate (0.5 ml per 100 g) prior to surgery. BSMP samples were dried at 60°C in a vacuum for 5 hours and sterilized by UV irradiation for 3 hours and were then placed into transparent glass bottles. Each rat tail measuring 16 cm in length was cut 6 cm from the tip using surgical scissors. Each wound section was covered with BSMPs directly to control bleeding with slight pressure. The cessation of blood flow was timed. A gauze sponge served as a control condition in this study. At the end of the experiment, the rats were euthanized using an overdose of anesthesia.

### 2.7. Statistical Analysis

Data points are expressed as the means ± standard deviations. Where suitable, data were analyzed using ANOVA single factor analyses to demonstrate differences between groups. Differences were considered statistically significant at *p* < 0.05.

## 3. Results

### 3.1. BSMP Characterization

**Figure 1:**
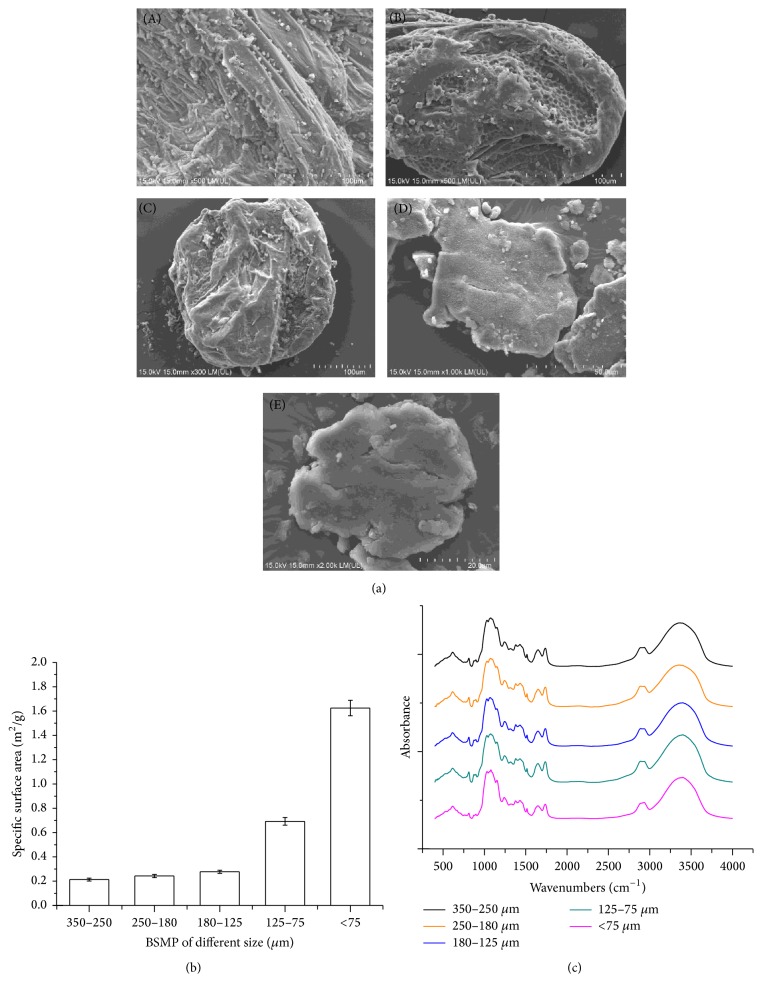
(a) SEM images of surface structures of BSMPs of various sizes ((A) 350–250 *μ*m; (B) 250–180 *μ*m; (C) 180–125 *μ*m; (D) 125–75 *μ*m; (E) <75 *μ*m;). (b) Effects of particle size on specific surface area. (c) The FTIR of BSMPs of different sizes.

The SEM images shown in Figure 1(a) present morphological characteristics of the BSMPs (350–250, 250–180, 180–125, and <75 *μ*m). As particle sizes decreased, surface features of the BSMPs became smoother. The specific surface areas of BSMPs of different sizes are shown in Figure 1(b). Surface areas ranged from 102.602 to 366.878 m^2^/g. As particle sizes decreased, surface areas increased. The specific surface of BSMPs with a particle size of <75 *μ*m was higher than that of other BSMPs. The FTIR spectrum of the BSMP samples from 400 to 4000 cm^−1^ is shown in Figure 1(c), and the results show that no new chemical bonds formed in the BSMPs. The wavenumbers of functional groups of the BSMP samples are given in Table 1. In the “fingerprint” region, the spectra are very complex. As Figure 1(c) and Table 1 show, the overall spectral profiles of BSMPs of different sizes were almost uniform.

### 3.2. Blood/BSMP Aggregation

**Figure 2:**
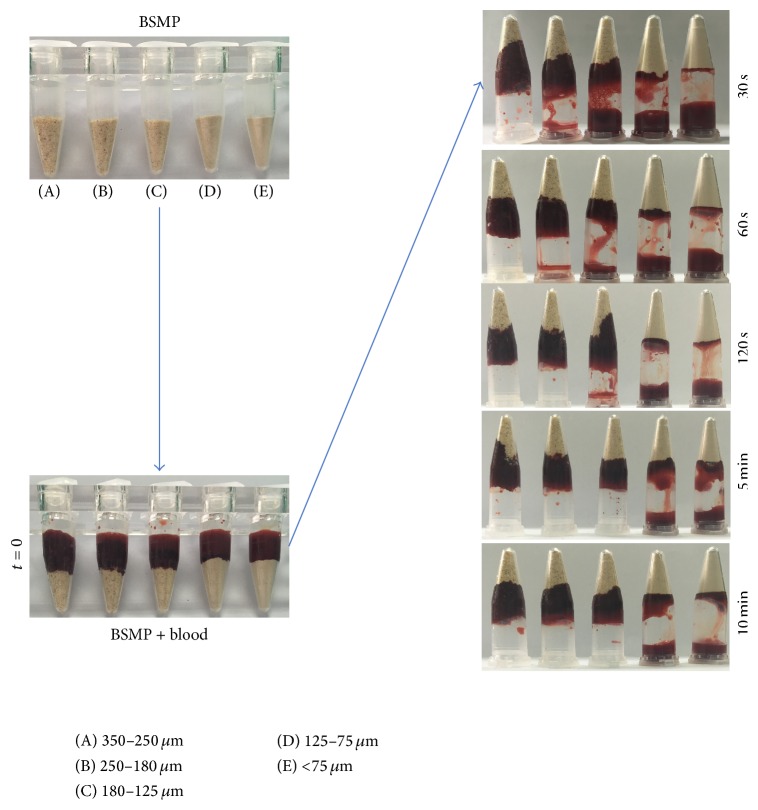
Macroscopic view of BSMP (350–250, 250–180, 180–125, and <75 *μ*m) Stopper-induced formation of blood/BSMP aggregates in the anticoagulant blood.

Coagulation time was evaluated from plastic vials to elucidate any direct effects on coagulation in vitro. When anticoagulant blood was added to the BSMPs, a coagulum formed significantly faster in the BSMPs (350–250 *μ*m) than in the other groups (Figure 2). As particle sizes decreased, BSMP capacities to absorb blood weakened. A delay in blood coagulation was observed with a decrease in BSMP particle size. Even in cases of delay, an aggregation quickly formed between a fraction of the blood and particles.

### 3.3. The Internal Structure of Blood/BSMP Aggregation

**Figure 3:**
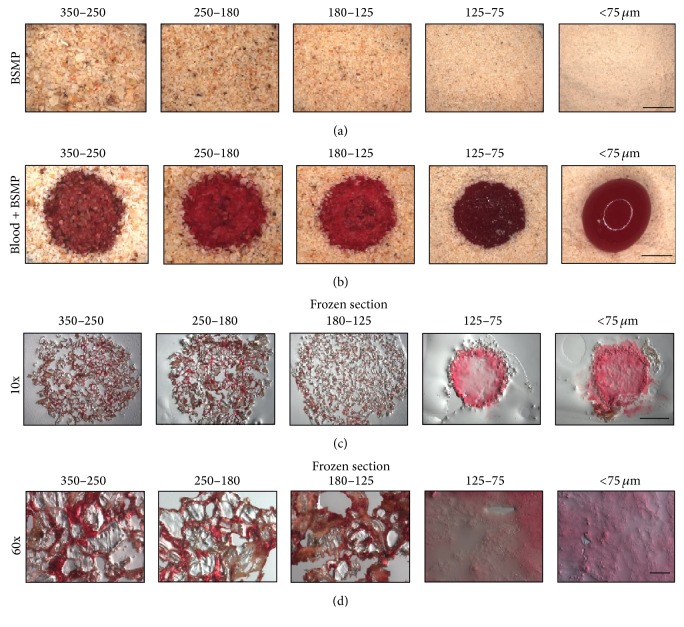
The internal structure of blood/BSMP aggregation. (a) Photographs of the BSMPs under a stereoscope (Scale Bar = 2 mm). (b) Photographs of the BSMPs and anticoagulant blood under a stereoscope (Scale Bar = 2 mm). (c) Photographs of frozen sections of the blood/BSMP aggregation (Scale Bar = 2 mm). (d) Photographs of frozen sections of the blood/BSMP aggregation (Scale Bar = 100 *μ*m).

To further study the hemostatic mechanisms of BSMPs, we observed blood/BSMP aggregation formation under a stereoscope (Figures 3(a) and 3(b)). BSMPs (350–250, 250–180, and 180–125 *μ*m) in contact with anticoagulant blood formed visible aggregations, but BSMPs (125–75 and <75 *μ*m) did not form blood/BSMP aggregations after being in contact with anticoagulant blood, and anticoagulant blood even gathered on surfaces of the BSMPs (<75 *μ*m) (Figure 3(b)). Thus, BSMP particle sizes had a crucial influence on blood/BSMP aggregation formation. The internal structure of the blood/BSMPs (350–250, 250–180, and 180–125 *μ*m) is shown in Figures 3(c) and 3(d). BSMP particles gathered via anticoagulant blood (Figures 3(c) and 3(d)). As BSMP particle sizes decreased, the degree of aggregation between BSMP particles declined. Blood indeed gathered on the surfaces of the BSMPs (125–75 and <75 *μ*m) (Figures 3(b), 3(c), and 3(d)).

### 3.4. Texture Analysis of Blood/BSMP Aggregations

**Figure 4:**
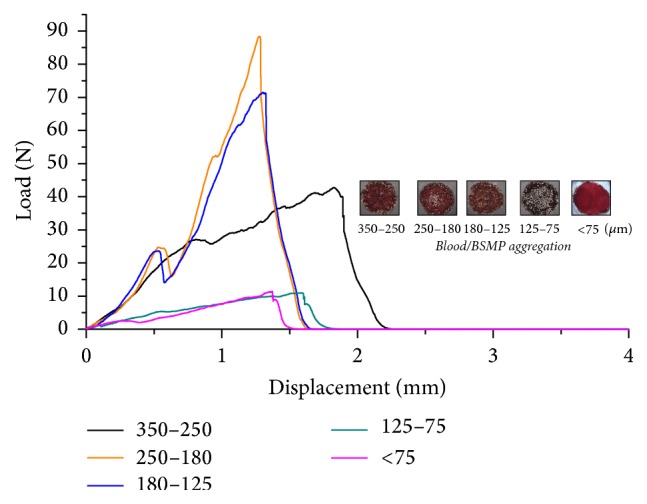
Texture analysis diagram of the blood/BSMP (350–250, 250–180, 180–125, and <75 *μ*m) aggregations.

**Figure 5:**
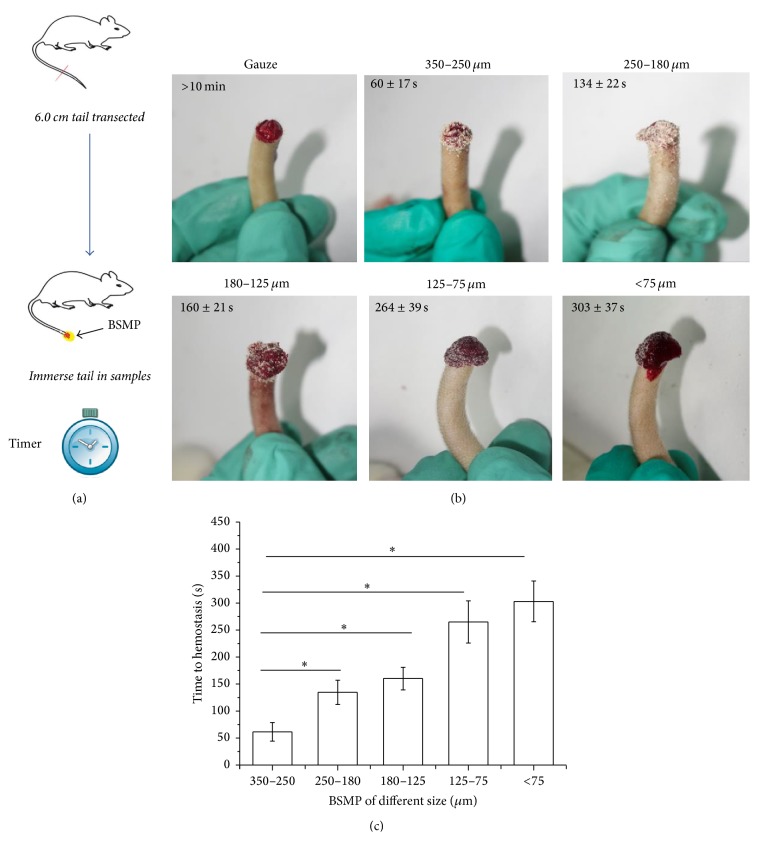
The hemostatic effect of the BSMPs evaluated by the rat tail amputation model. (a) A segment of each rat tail was amputated transversely, and the cut ends were immediately immersed in the BSMPs. (b) Photograph of tail amputation after short-term BSMP application (350–250, 250–180, 180–125, 125–75, and <75 *μ*m) showing that bleeding has stopped and 10 min application of gauze showing continued bleeding. (c) Time to hemostasis for the same injury models (*n* = 5 for each group, ^*∗*^*p* < 0.05).

A texture analysis of the blood/BSMP (350–250, 250–180, 180–125 125–75, and <75 *μ*m) aggregations is shown in Figure 5. Hardness values of the blood/BSMP (180–15 and <75 *μ*m) aggregations were similar at roughly 10 N with no obvious signs of brittleness. Compared to blood/BSMP (125–75 and <75 *μ*m) aggregations, the stress curve of blood/BSMP (250–180 and 180–125 *μ*m) aggregations was found to be analogous, and the hardness of blood/BSMP (250–180 and 180–125 *μ*m) aggregations reached a maximum value. As is shown in Figure 4, while the fragmentation of blood/BSMP (350–250) aggregation required constant force, squeeze forces did not increase rapidly. Compared to the other groups, measurements of the blood/BSMP (350–250) aggregation showed apparent toughness owing to its internal structure.

### 3.5. Rat Tail Amputation

Initial efficacy studies were performed on tail amputation rat models (Figure 5(a)). As particle sizes decreased, hemostatic effects of BSMPs were attenuated as shown in Figures 5(b) and 5(c). After ruling out individual differences, BSMPs (350–250 *μ*m) rapidly controlled bleeding after 60 s and wounds clotted after applying only a small number of BSMPs (350–250 *μ*m) whereas the gauze control stopped bleeding but did not promote coagulation even after 10 min. The value for the gauze sponge conditions is therefore not represented in Figure 5(c). BSMPs in contact with a bleeding wound formed a visible aggregate and a rapid sealant at the surface of each wound, allowing for hemostasis to be reached quickly as shown in Figure 5(b).

## 4. Discussion

*B. striata* is a folkloric herb of the Orchid family that has been widely used in Traditional Chinese Medicine (TCM) as natural styptic powder for treating lung and stomach bleeding [10]. Our study results show that* B. striata* Micron Particles (BSMPs) spur hemostatic modes of action by forming a visible particle/blood aggregate as a physical barrier that gives rise to homeostasis. Compared to other hemostatic materials, BSMPs serve as an inexpensive, natural, and promising alternative.

The present study shows that BSMP size ranges are likely a key factor affecting hemostatic outcomes. With BSMP preparation, as particle sizes decreased, the surface structures of BSMPs (125–75 and <75 *μ*m) changed, resulting in hemostatic inefficiency (Figure 1(b)). Compared to other groups, the special surface structures of BSMPs (350–250 *μ*m) enable them to promote blood/particle aggregation and to form rapid sealants on wound surfaces to achieve rapid hemostasis. Recent studies have shown that* B. striata* contains numerous polysaccharides that have been identified as major active components responsible for the various biological effects [12]. Furthermore, bioactivity evaluations have revealed hemostatic activities of* B. striata* polysaccharides [13]. Polysaccharides on BSMP surfaces are a key factor affecting the hemostatic efficiency of BSMPs. We hypothesized that when BSMPs come into contact with blood, red blood cell aggregation and adhesion on polysaccharide surfaces form blood/BSMP aggregations that spur rapid hemostasis (Figure 3(d)).

The* Bletilla striata* polysaccharide is also known to protect against* Staphylococcus aureus *[14], to control inflammatory responses, and to accelerate wound closure, presenting potential applications for wound healing [15]. A novel water-soluble polysaccharide,* Bletilla striata* polysaccharide b (BSPb), has been isolated from* Bletilla striata*. BSPb was found to possess antioxidative stress and to offer anti-inflammatory functions against Ang II-induced ROS generation and proinflammatory cytokines activation [16]. BSMPs, in addition to stopping bleeding, offer anti-inflammatory properties and promote wound healing.

To improve the hemostasis efficiency of* Bletilla striata*, future studies will involve preparing* Bletilla striata* polysaccharide hydrogel particles via blood aggregation as a hemostatic agent and comprehensive investigations of their hemostasis mechanisms [17]. For single injury models, hydrogel aggregate formation at the injury site can control bleeding.

## 5. Conclusion

The present study demonstrates that the facile production of BSMPs can show promise as an effective hemostatic agent. Anticoagulant blood/particle aggregations, internal structures of blood/BSMP aggregations examined under a stereoscope and texture analyses of blood/BSMP aggregations were used to predict the in vivo behaviors of BSMPs of different sizes. In vitro, an aggregate was formed in a fraction of the blood and BSMPs, forming a physical barrier to further blood loss. As the particle sizes of BSMPs decreased, the degree of aggregation declined. In vivo, hemostatic capacities of BSMPs of different sizes showed a decrease in the time to hemostasis in animal injury model. The hemostasis results of 350–250 *μ*m BSMPs were found to be the most efficient of the five different sizes of BSMPs tested. To our knowledge, this is the first report on hemostatic mechanisms of BSMPs and the first efficacy study on BSMPs, which upon coming into contact with a bleeding surface form aggregations or sealants at wound surfaces that quickly spur hemostasis. This physical mechanism is not dependent on the body's physiological mechanisms and is therefore effective even for patients with coagulation disorders. Compared to other hemostatic materials such as chitosan hemostatic materials, zeolite, and mesoporous silica, BSMPs present many advantages (ease of preparation, low cost, long shelf life, and nontoxicity). BSMPs can be used as hemostatic with practical hemostatic mechanisms for treating trauma-related bleeding.

## Supplementary Material

 Schematic illustration of *Bletillastriata*via grinding as a hemostatic agent for promoting rapid blood aggregation.

## Figures and Tables

**Table 1:** Major absorptions in IR spectra of BSMPs of different sizes.

BSMP (*μ*m)					Assignment
>250	250–180	180–125	125–75	<75	
Frequency (cm^−1^)					
3399	3399	3399	3387	3387	O–H and N–H group stretching
2890	2890	2890	2890	2890	C–H stretching
1735	1736	1736	1736	1736	C=O stretching
1647	1647	1647	1650	1648	COO– stretching and C=C aromatic skeletal vibration
1514	1514	1514	1514	1514	Aromatic skeletal stretching
1431	1431	1429	1431	1429	CH3 and CH symmetric bending
1377	1379	1379	1379	1379	CH3 and CH symmetric bending
1319	1317	1315	1315	1316	C–N stretching
1239	1243	1244	1243	1242	C–O stretching
1151	1150	1150	1150	1150	C–O stretching
1078	1075	1065	1075	1075	C–O stretching
1031	1030	1030	1031	1031	C–O stretching
896	895	895	895	895	C–H stretching out of plane of aromatic ring
811	811	811	811	811	C–H stretching out of plane of aromatic ring
614	614	614	614	614	O–H bending

## References

[1] Champion H. R., Bellamy R. F., Roberts C. P., Leppaniemi A. (2003). A profile of combat injury. *Journal of Trauma—Injury, Infection and Critical Care*.

[2] Collen D. (1980). On the regulation and control of fibrinolysis. Edward Kowalski Memorial Lecture. *Thrombosis and Haemostasis*.

[3] de Gaetano G. (2001). Historical overview of the role of platelets in hemostasis and thrombosis. *Haematologica*.

[4] Ward K. R., Tiba M. H., Holbert W. H. (2007). Comparison of a new hemostatic agent to current combat hemostatic agents in a swine model of lethal extremity arterial hemorrhage. *Journal of Trauma - Injury, Infection and Critical Care*.

[5] Gabay M., Boucher B. A. (2013). An essential primer for understanding the role of topical hemostats, surgical sealants, and adhesives for maintaining hemostasis. *Pharmacotherapy*.

[6] Achneck H. E., Sileshi B., Jamiolkowski R. M., Albala D. M., Shapiro M. L., Lawson J. H. (2010). A comprehensive review of topical hemostatic agents: efficacy and recommendations for use. *Annals of Surgery*.

[7] He X., Wang X., Fang J. (2017). Bletilla striata: medicinal uses, phytochemistry and pharmacological activities. *Journal of Ethnopharmacology*.

[8] Kong J.-M., Goh N.-K., Chia L.-S., Chia T.-F. (2003). Recent advances in traditional plant drugs and orchids. *Acta Pharmacologica Sinica*.

[9] Zheng C., Feng G., Liang H. (1998). Bletilla striata as a vascular embolizing agent in interventional treatment of primary hepatic carcinoma. *Chinese Medical Journal*.

[10] Hung H.-Y., Wu T.-S. (2016). Recent progress on the traditional Chinese medicines that regulate the blood. *Journal of Food and Drug Analysis*.

[11] Yu L., Nie X., Pan H., Ling S., Zhang D., Bian K. (2011). Diabetes mellitus ulcers treatment with Bletilla striata polysaccharide. *Zhongguo Zhongyao Zazhi*.

[12] Wang Y., Liu J., Li Q., Wang Y., Wang C. (2015). Two natural glucomannan polymers, from Konjac and Bletilla, as bioactive materials for pharmaceutical applications. *Biotechnology Letters*.

[13] Cui X., Zhang X., Yang Y., Wang C., Zhang C., Peng G. (2016). Preparation and evaluation of novel hydrogel based on polysaccharide isolated from *Bletilla striata*. *Pharmaceutical Development and Technology*.

[14] Li Q., Li K., Huang S.-S., Zhang H.-L., Diao Y.-P. (2014). Optimization of extraction process and antibacterial activity of bletilla striata polysaccharides. *Asian Journal of Chemistry*.

[15] Luo Y., Diao H., Xia S., Dong L., Chen J., Zhang J. (2010). A physiologically active polysaccharide hydrogel promotes wound healing. *Journal of Biomedical Materials Research. A*.

[16] Yue L., Wang W., Wang Y. (2016). Bletilla striata polysaccharide inhibits angiotensin II-induced ROS and inflammation via NOX4 and TLR2 pathways. *International Journal of Biological Macromolecules*.

[17] Behrens A. M., Sikorski M. J., Li T., Wu Z. J., Griffith B. P., Kofinas P. (2014). Blood-aggregating hydrogel particles for use as a hemostatic agent. *Acta Biomaterialia*.

