# Supplementary figures and images for: Bletilla striata Micron Particles Function as a Hemostatic Agent by Promoting Rapid Blood Aggregation
Source: Evid Based Complement Alternat Med. 2017 Mar 12;2017:5820405. doi: 10.1155/2017/5820405 (PMC5366200; doi:10.1155/2017/5820405)

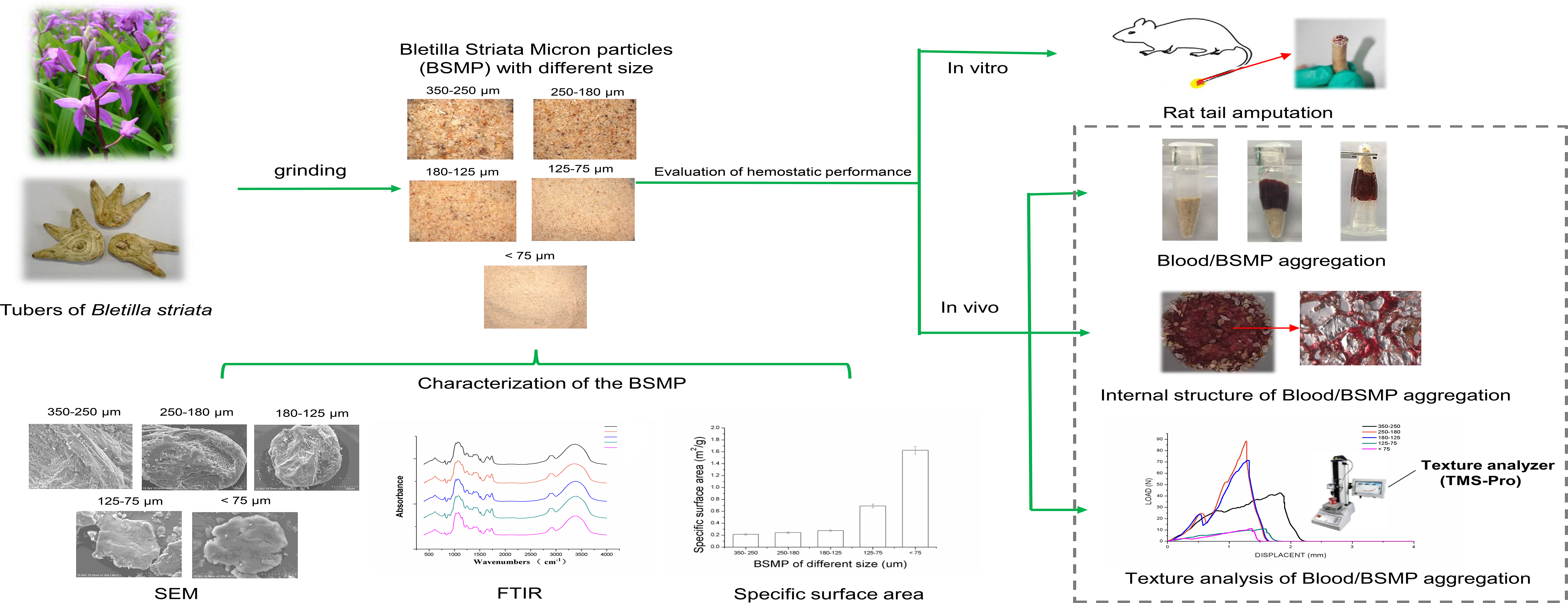

Supplement: Supplementary file 1 — Schematic illustration of Bletillastriatavia grinding as a hemostatic agent for promoting rapid blood aggregation. [file 5820405.f1.tif]
